# Designing Ago2-specific siRNA/shRNA to Avoid Competition with Endogenous miRNAs

**DOI:** 10.1038/mtna.2014.27

**Published:** 2014-07-15

**Authors:** Hongming Ma, Junli Zhang, Haoquan Wu

**Affiliations:** 1 Department of Biomedical Sciences, Center of Excellence in Infectious Disease Research, Paul L. Foster School of Medicine, Texas Tech University Health Sciences Center, El Paso, Texas, USA.

**Keywords:** Ago2, Dicer, miR-451, shRNA

## Abstract

Relatively large amounts of transfected siRNA can compete for Ago proteins and thus compromise endogenous miRNA function, potentially leading to toxicities. Here, we show that shRNA can also perturb endogenous miRNA function similarly. More importantly, we also show that the problem can be solved by designing shRNAs in the context of pre-miR-451 structure with completely complementary stem, which significantly improves the Ago2 specificity. This shRNA was shown to be Ago2-specific, and maintain target-silencing ability while avoiding competition with endogenous miRNAs by not competing for Agos 1, 3, and 4. We conclude that modified pre-miR-451 structure provides a general platform to design shRNAs that significantly reduce perturbation of miRNA function.

## Introduction

In mammals, the canonical miRNA biogenesis pathway is initiated by the Drosha–DGCR8 complex, which processes long-primary miRNAs (pri-miRNAs) into short pre-miRNAs for further processing by Dicer into mature miRNA duplexes. One or both strands of the duplex are loaded into the RNA-induced silencing complex (RISC) to suppress target genes.^1,2^ This endogenous miRNA-processing machinery is the basis for why exogenously introduced siRNA/shRNA can function in cells. siRNAs and shRNAs mimic miRNA biogenesis intermediates at different stages—siRNAs are mainly mimics of miRNA duplexes while conventional shRNAs are mimics of pre-miRNAs. To be functional, these siRNAs and shRNAs have to be processed by the miRNA processing system and finally loaded into RISC.

The key components of RISC are the Argonaute (Ago) proteins.^3^ In mammals, there are four Ago proteins, and the exact function of each is not clear. For siRNAs or shRNAs, previous studies have suggested that Ago2 is the “slicer” that cleaves the target mRNA, and is therefore, the predominant contributor to siRNA repression.^4,5^ However, it is also reported that translational inhibition mediated by the other three Ago proteins (Ago1, 3, and 4) also significantly contributes to siRNA repression.^6^ In addition to different repression mechanisms, the loading of siRNA or miRNA can also differ between Ago2 and the other Ago proteins because Ago2 can cleave the passenger strand, which is removed by C3PO (component 3 promoter of RISC, a complex of Translin and Trax);^7,8^ while Ago1, 3, and 4 cannot cleave the passenger strand, and must load siRNA or miRNA using the “bypass mechanism”.^9^ Thus, Ago2 is distinct from the other three Ago proteins in its slicer function.

Conventional shRNA is generally believed to be processed by Dicer into a duplex and loaded into RISC. However, it has been shown that the functionality of short shRNAs with a stem length of 19 bp or less do not require Dicer processing;^10,11^ and how they are processed and loaded into RISC has been unclear. However, recent reports on miR-451 biogenesis have now solved this puzzle. It turns out that pre-miR-451, which has an 18-nt long stem and a 4-nt long terminal loop, does not require Dicer processing. Instead, it is loaded directly into Ago2 and cleaved at position 10 of the passenger strand to generate 30-nt intermediate products, which are further trimmed by an unknown nuclease to generate the mature miR-451.^12,13,14^ Thus, miR-451 biogenesis is Dicer-independent, suggesting that short shRNAs with a similar structure will also be processed directly by Ago2, and this has been confirmed experimentally by multiple reports.^12,13,14,15,16,17,18^ These Ago2-specific shRNAs will have reduced off-target effects induced by passenger strand loading because the passenger strand is cleaved by Ago2 during its maturation.^15^

Here, using mouse embryonic stem (ES) cells expressing only individual Ago proteins, we showed that Ago2 was the major executor of siRNA-mediated silencing when the target sites were fully complementary to the siRNA; but showed similar functionality to the other Ago proteins in miRNA-mediated target gene repression when the target sites were not perfectly matched with the miRNA, and therefore Ago2-specific siRNA/shRNA can avoid perturbing endogenous miRNA function. We also evaluated methods of designing Ago2-specific siRNA/shRNA and studied how to improve Ago2 specificity in this system.

## Results

### Ago2 is the major executor in siRNA-mediated silencing but not in miRNA function

**Figure 1:**
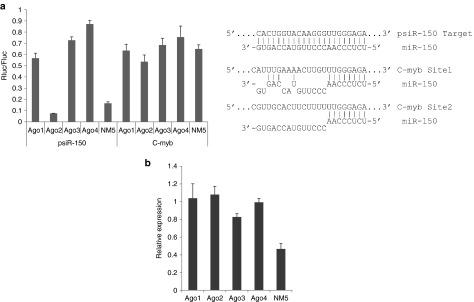
**Ago2 is the predominant enzymatic contributor to repression of siRNA targets with a fully complementary target sequence, but its contribution is similar to the other three Ago proteins in the case of miRNA targets**. (**a**) miR-150 functionality in ES cells expressing individual Ago proteins. NM5 is the parental ES cell line, which expresses all Ago proteins.^19^ psiR-150 is a pCHECK2 vector containing a 2× repeat of a target sequence that is fully complementary to miR-150 and inserted as an siRNA-like target in the 3′ UTR of the Renilla luciferase gene. C-myb represents a psiCHECK2 vector containing the full-length 3′ UTR of the C-myb gene, which is one of the strongest natural targets of miR-150. The base-pairing between miR-150 and target sites is shown. The dual-luciferase assay was performed 24 hours after cotransfection of pri-miR-150 with the indicated reporter vector. The ratio of Renilla luciferase (Rluc, reporter) to firefly luciferase (Fluc, internal control) normalized to the negative control (identical to pri-miR-150 but with scrambled mature miR-150 sequences) is shown. The experiments were performed in triplicate. (**b**) The relative expression level of miR-150 measured by real-time PCR and normalized to the eGFP expression level. The eGFP gene is contained in the same vector (pLL3.7) used to express miR-150. Error bar, 1 S.D.

Knockout of all Ago genes in mouse ES cells followed by reconstitution with individual Agos is an excellent strategy for dissecting the functionality of individual Ago proteins.^19^ Previously, we tested the loading of siRNA or miRNA-like duplexes into different Ago proteins in these cells and showed that all four Ago proteins can load both siRNA and miRNA structured oligos to generate active RISC similarly.^20^ Here, we wanted to study the functionality of different Ago proteins in inhibiting siRNA or miRNA targets in this system. In general, siRNAs were designed to perfectly match the target sites, while miRNAs did not perfectly match the target sequence. C-myb is a validated natural target of miR-150,^21^ which has two typical target sites; thus, we used the C-myb full-length 3′ UTR as a miR-150 miRNA target. psiR-150 was designed as a siRNA target with two sequence repeats fully complementary to miR-150. The target sequences were inserted into the 3′ UTR of the *Renilla* luciferase gene of psiCHECK2 vector and cotransfected with a plasmid expressing pri-miR-150. The repression efficacy was determined by measuring luciferase activity. As shown in **Figure 1a**, the functionality of miR-150 is dramatically higher with Ago2 than with the other three Ago proteins in repressing the siRNA target, psiR-150, while the functionality is similar to the other three Ago proteins in repressing the miRNA target, C-myb (**Figure 1a**). It has been shown that Ago protein expression level is similar in these cells,^19^ the functionality difference seen in siRNA targets should not be the results of Ago2 expression level. Thus, to exclude the possibility that a loading difference caused the level of miR-150 difference and therefore, functionality difference, we performed Q-PCR, and the results showed that the level of miR-150 is similar in all four Ago protein-expressing cells (**Figure 1b**), which is consistent with the functionality of miR-150 in C-myb target (**Figure 1a**). Thus, consistent with previous reports, our results clearly show that, for fully complementary siRNA targets, Ago2 is the major executor, while the contribution of the other three Ago proteins is insignificant, although it has been reported that their translational inhibition also significantly contributes to siRNA repression.^6^ However, the contribution of Ago2 in repressing miRNA targets is similar to the other three Ago proteins.

To summarize, Ago1, 3, and 4 can effectively repress miRNA targets without Ago2, while Ago2 can efficiently repress siRNA targets without Ago1, 3, and 4. Thus, Ago2-specific siRNA/shRNA might exhibit full functionality and, at the same time, minimize the disturbance of endogenous miRNA function.

### The Ago2 specificity of shRNAs with a miR-451-like structure

**Figure 2:**
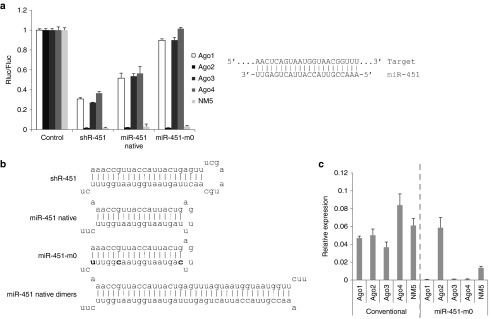
**The Ago2 specificity of shRNAs with a miR-451-like structure**. (**a**) The functionality of shRNAs with different structures in ES cells expressing individual Ago proteins was assessed as in **Figure 1a**. All shRNAs were driven by the U6 promoter, and the exact sequences are listed in **Supplementary Table S1**. The base-pairing between miR-451 and target site is shown. The shRNA structures are shown in (**b**). The mutated nucleotides in miR-451-m0 are marked bold and the dimer structure of native miR-451 is shown in the bottom. (**c**) The relative expression of miR-451 was assessed as in **Figure 1b**.

It was previously shown that a miR-451-like structure with a short stem of 19 nt or less can be processed by Ago2 and should be Ago2-specific.^12,13,14,15,16,17,18^ ES cells expressing only individual Ago proteins are good tools to evaluate the Ago2 specificity of miR-451-like shRNAs. Like shRNA, the pre-miR-451 was expressed by a U6 promoter. It is anticipated that the functionality of pre-miR-451 will be Ago2 specific. However, compared with conventional shRNA (shR-451 with a 22-nt stem and an 8-nt loop, as shown in **Figure 2b**), the pre-miR-451 (native) was also functional with Ago1, 3, and 4, although the activity was slightly lower (**Figure 2a**). We suspected that the reason might be that native pre-miR-451 can form dimers. Native miR-451 has one mismatch at position one and a G:U wobble pair at positions 6 and 18 (**Figure 2b**). With large numbers of transcripts generated from the U6 promoter, these transcripts have a high probability of forming dimers, which are much more stable (having lower ΔG). The dimers can be processed by Dicer into duplexes^22,23,24,25^ that can be loaded into Ago1, 3, or 4. We hypothesized that stabilizing the stem of native pre-miR-451 might reduce the propensity of forming dimers and therefore, improve the Ago2 specificity. We changed the nucleotides at positions 23, 35, and 40 to make a miR-451 with a completely complementary stem (miR-451-m0, **Figure 2b**). As shown in **Figure 2a**, this stem-stabilized variant of miR-451 appeared to be completely Ago2-specific—no functionality was observed in Ago1, 3, and 4, while the functionality was maintained in Ago2. To further confirm that miR-451-m0 is Ago2-specific, we determined the mature miR-451 level in the cells expressing individual Ago proteins. As shown in **Figure 2c**, for shR-451 (with a conventional shRNA design), the mature miR-451 levels are similar with all four Ago proteins, while for miR-451-m0 (with a stabilized stem), a high level of miR-451 is observed only in Ago2-expressing cells and not with Ago1, 3, and 4, suggesting that miR-451-m0 is indeed Ago2-specific.

### Ago2-specific shRNA can avoid the interference of endogenous miRNA function caused by shRNA expression

It has been reported that transfection of siRNA can globally perturb gene expression because of the impaired effectiveness of endogenous miRNA repression probably caused by the competition for Ago proteins between the large number of transfected siRNAs and endogenous miRNAs.^26^ Unlike siRNA that is transfected with relatively large amounts, shRNA is generally expressed with a vector to generate siRNA continuously in cells. To achieve strong target repression, shRNA is commonly expressed with strong pol III promoter, such as U6, to generate large numbers of mature siRNAs. Previously, to determine the mature siRNA sequence generated from shRNA, we transfected a conventional shRNA into 293FT cells and cloned the small RNAs generated. To our surprise, just after 24 hours of transfection, the total reads of mature siRNA sequence generated from the shRNA were even more than the total reads of all endogenous miRNAs together (data not shown), suggesting that the large numbers of siRNAs generated from shRNAs might also compete for the Ago proteins and compromise the repression function of endogenous miRNAs. Ago2-specifc shRNA might avoid the competition for Ago 1, 3, and 4 and therefore mitigate the interference of endogenous miRNA function.

**Figure 3:**
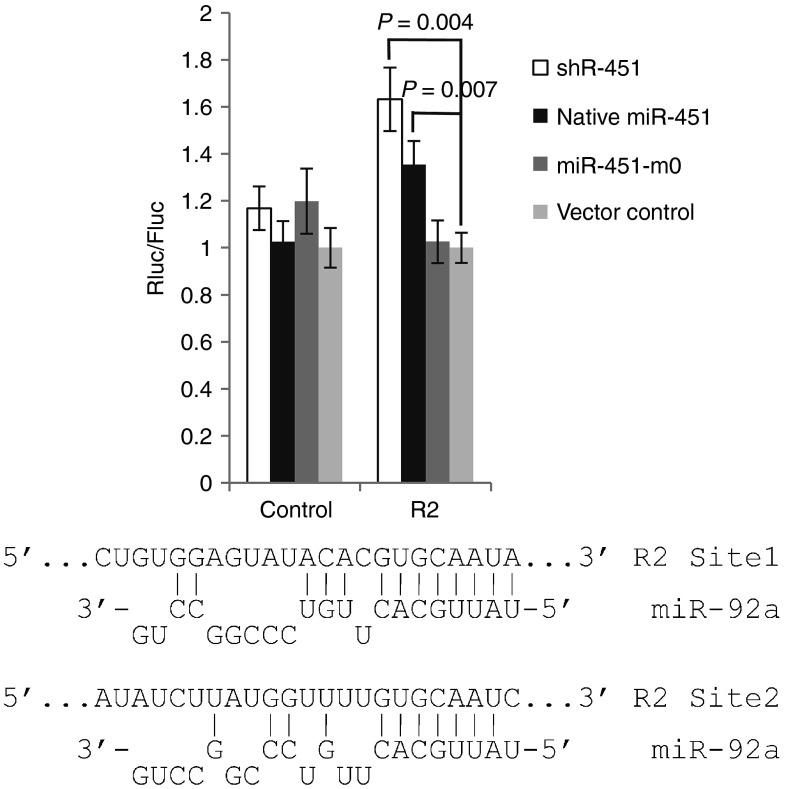
**Ago2-specific shRNA can mitigate the interference of endogenous miRNA function caused by shRNA expression**. R2 contains a fragment from 3′ UTR of *Phllp2* gene (from 2457 bp to 3396 bp) that has two target sites for miR-92a,^27^ which is one of the most abundant miRNAs expressed in 293FT cells. The base-pairing between miR-92a and target sites is shown. The control plasmid contains a fragment from 3′ UTR of the same gene (from 1071bp to 1565bp), which contain only one target site for miR-137, whose expression level is very low in 293FT cells (thousands times lower than miR-92a, data not shown). The target sites were inserted into the 3′ UTR region of the Renilla luciferase reporter gene in psiCHECK2 vector and cotransfected with indicated shRNAs into 293FT cells. The luciferase activity was measured 48 hours later. The ratio of Renilla luciferase (Rluc, reporter) to firefly luciferase (Fluc, internal control) normalized to the vector control is shown. The experiments were performed in triplicate.

To test the hypothesis, we determined the effect of shRNAs with different structures on the repression function of endogenous miRNA. To evaluate the endogenous miRNA repression function, we choose a validated target gene *Phllp2* for miR-92a,^27^ which is one of the most abundant miRNAs expressed in 293FT cells (data not shown). A fragment of 3′ UTR of *Phllp2*, which contains two conserved target sequences of miR-92a, was inserted into the 3′ UTR region of the Renilla luciferase reporter gene in psiCHECK2 vector.^27^ The control plasmid contains a fragment from 3′ UTR of the same gene (from 1071 to 1565bp) that contains only one target site for miR-137, whose expression level is very low in 293FT cells (thousands times lower than miR-92a, data not shown). We cotransfected different shRNAs with the constructs separately and measured the luciferase activity to evaluate the repression of endogenous miRNA functionality. As shown in **Figure 3**, for R2, the repression of the target is maintained at the same level for Ago2-specific shRNA miR-451m0 compared with the vector control, while the repression is significantly compromised for the conventional shR-451, and also for the native miR-451 to a lesser extent, suggesting that conventional shRNA can significantly compromise the function of endogenous miRNA while Ago2-specific shRNA can avoid the interference caused by shRNA expression. The data suggest that shRNA can compromise the function of endogenous miRNA and designing shRNAs with high Ago2 specificity, which have a pre-miR-451-like structure with completely complementary stem, can avoid perturbing endogenous miRNA repression functionality.

### The optimal stem length for Ago2-specific shRNA

**Figure 4:**
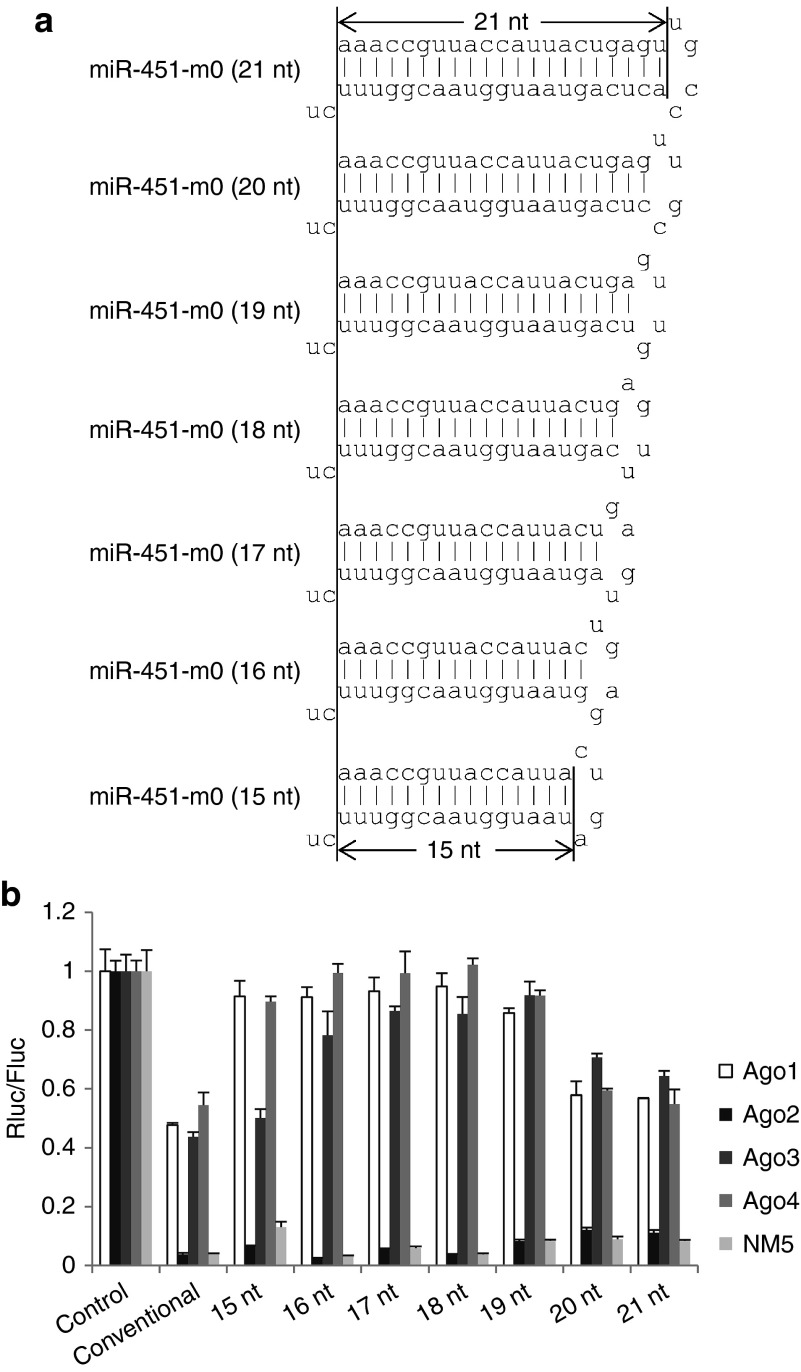
**The impact of stem length on Ago2 specificity**. (**a**) The structure of miR-451-m0 with indicated length. (**b**) The functionality of miR-451-m0 with the indicated stem lengths in ES cells expressing individual Ago proteins was assessed as in **Figure 1a**. The base-pairing between miR-451 and target site is the same as **Figure 2a**.

Native miR-451 has a unique structure, with an 18-nt stem and 4-nt terminal loop. Previously, it was reported that stem length can affect shRNA functionality.^11,15,16,28^ To determine the optimal stem length for Ago2-specific shRNA, we tested the impact of stem length on functionality and specificity. Using stem-stabilized miR-451 (miR-451-m0) as our starting point, we designed miR-451 variants with stem lengths ranging from 15–21 nt. As shown in **Figure 4**, miR-451-m0 with 16-, 17-, and 18-nt stems showed excellent Ago2 specificity—having good functionality with Ago2 and almost no functionality with Ago1, 3, and 4—while miR-451-m0 with 15- and 19-nt stems showed decreased Ago2 specificity, and miR-451-m0 with 20- and 21-nt stems had completely lost Ago2 specificity. Thus, a 16- to 18-nt stem length appears to be optimal.

### Ago2-specific shRNAs have different sequence preferences and appear to be more potent than conventional shRNAs

**Figure 5:**
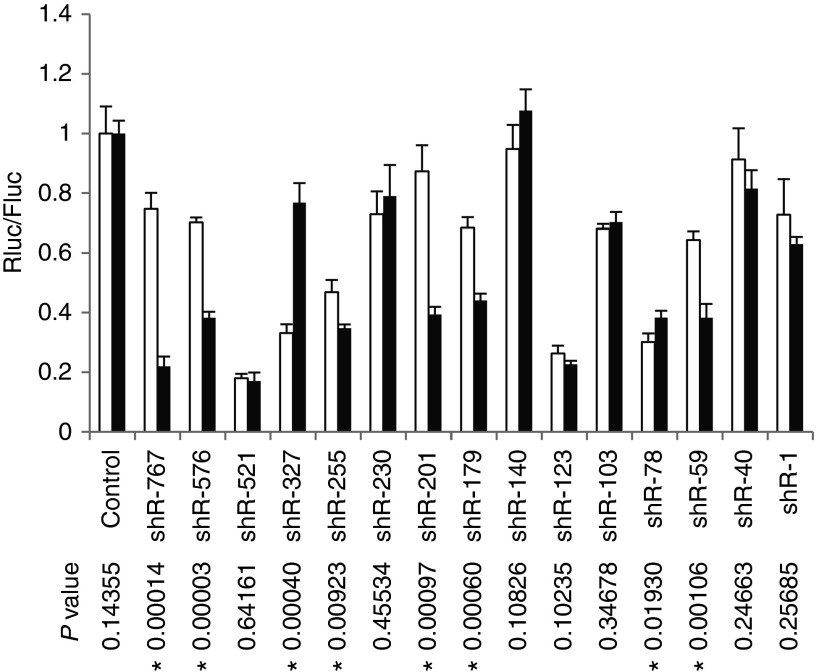
**The functionality of Ago2-specific shRNAs compared with conventional shRNAs was assessed as in Figure 1a but in the 293FT cell line**. The shRNAs targeting the *Renilla* luciferase gene were designed as conventional shRNAs (white bars, with a 20-nt stem and large terminal loop) or with a miR-451-m0-like structure (black bars). *P* values are shown for each pair of shRNAs. * indicate statistical significance.

Sequence preference has been well documented for conventional shRNA—some sequences show high functionality while others show little or no functionality. Conventional shRNAs are processed by Dicer into a duplex ~22 nt in length, and one of the strands is loaded into Ago proteins. Ago2-specific shRNAs with a shorter stem are processed differently—they are loaded into Ago2 directly and cleaved by Ago2. Because of the different processing pathways, we hypothesized that Ago2-specific shRNAs might have different sequence preferences than conventional shRNAs. Thus, we randomly selected 15 target sites in the *Renilla* luciferase gene and designed 15 pairs of shRNAs, either conventional or Ago2-specific (miR-451-m0 structure). Conventional shRNAs were designed to have a structure with a 20-nt stem and a 17-nt terminal loop, while Ago2-specific shRNAs were designed with a miR-451-like structure (18-nt stem and a 4-nt terminal loop). The functionality of these 15 pairs of shRNAs was determined in 293FT cells. As shown in **Figure 5**, eight pairs of shRNAs showed significantly different functionality, suggesting that Ago2-specific shRNAs have different sequence preferences than conventional shRNAs. Interestingly, in six out of eight cases, Ago2-specific shRNAs showed significantly higher functionality than conventional shRNAs, indicating that Ago2-specific shRNAs have a greater chance of high potency than conventional shRNAs.

### Design of Ago2-specific siRNA

**Figure 6:**
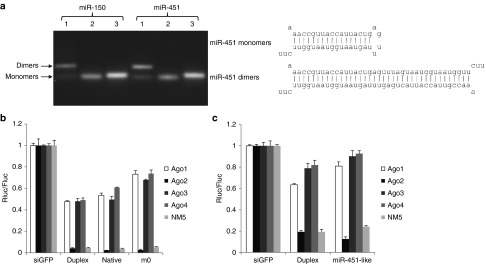
**Ago2 specificity of miR-451-like siRNAs**. (**a**) miR-150 and miR-451 RNA oligos with miR-451-like structures were separated in a 3% agarose gel. Lane 1: freshly dissolved oligos; lane 2: the oligos were diluted to 5 µmol/l followed by a quick cooling step; lane 3: siRNA duplex. (**b**) The functionality of miR-451 siRNA oligos was assessed as in **Figure 1a**, except that siRNAs and a siRNA-targeted GFP control were used. (**c**) The functionality of miR-150 oligos. Perfect match target sites are used in these experiments and the exact target sequences can be found in **Supplementary Table S2**.

A miR-451-like structure should also be applicable in designing Ago2-specific siRNA. However, we did not see any Ago2 specificity when we tested synthesized miR-451 RNA oligos, as both miR-451 and miR-150, which has a miR-451-like structure (18-nt stem and a 4-nt terminal loop), are functional in Ago1, 3, and 4, just like siRNAs with a duplex structure (data not shown). The most likely reason is that oligos with a miR-451-like structure can easily form dimers, which have a much lower ΔG than monomers and are therefore more stable (**Figure 6a**). As shown in lane 1 of **Figure 6a**, a majority of oligos formed dimers when freshly dissolved. By diluting the oligos to 5 µmol/l in combination with heat and quick-cooling steps, the oligos were transformed into monomers (lane 2 in **Figure 6a**), thus solving the problem. We could see a moderate increase in Ago2 specificity for both miR-150 and miR-451 (**Figures 5c** and **6b**); however, we did not achieve 100% Ago2 specificity as we saw in shRNAs driven by the U6 promoter. The reason might be that synthetic siRNA oligos were concentrated during the transfection process, and therefore siRNAs have a high probability of forming dimers. It is noteworthy to point out that, consistent with the results for shRNA, native miR-451 showed less specificity than miR-451 with stabilized stem (miR-451-m0, **Figure 6b**), supporting our previous conclusion that native miR-451 tends to form dimers, and stem-stabilizing variants like miR-451-m0 can effectively reduce this tendency.

**Figure 7:**
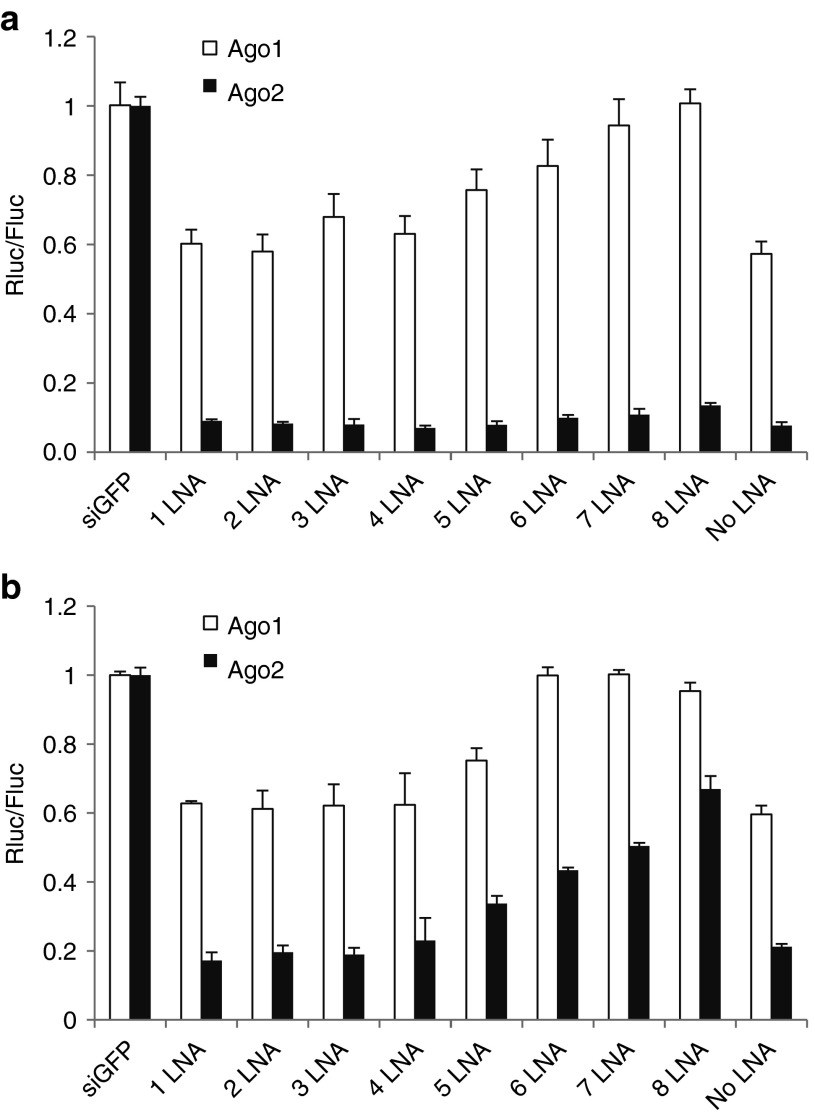
**Ago2 specificity of LNA-stabilized siRNA duplexes**. (**a**) The functionality of miR-451 siRNA duplexes stabilized with the indicated number of LNAs was assessed as in **Figure 6b**. (**b**) The functionality of miR-150 siRNA duplexes stabilized with the indicated number of LNAs. The target sites are the same as **Figure 6**.

Petri *et al*.^29^ reported that stabilizing the siRNA duplex by incorporating locked nucleic acids enables its selective loading into Ago2, leading to reduced off-target and elevated on-target effects. We therefore evaluated this approach in our system. We synthesized miR-451 and miR-150 passenger strands with different numbers of LNAs, as shown in **Supplementary Table S1**, and annealed the passenger strand with the guiding strand. After adding five or more LNAs, the stabilized miR-451 duplexes did indeed exhibit increased Ago2 specificity (**Figure 7**). However, while the functionality of the miR-451 duplex decreased only slightly after this addition (**Figure 7a**), it decreased significantly for the miR-150 duplex (**Figure 7b**). Thus, it appears that stabilizing the siRNA duplex by adding LNAs is a viable approach to designing Ago2-specific siRNAs; however, the impact on functionality is dependent on the siRNA sequence.

## Discussion

Although Ago2 slicer activity is known to be key for siRNA-mediated target degradation, its role in miRNA mediated target repression vis-à-vis other Agos and the role of other Agos in repression of fully complementary targets is not totally clear. It has been reported that Ago1, 3, and 4 can also make substantial contribution to the repression of fully complementary targets.^6^ Using mouse ES cells expressing only individual Ago proteins, we showed that Ago2 has dramatically higher functionality in repressing fully complementary targets (siRNA targets) compared with Ago1, 3, and 4 (**Figure 1a**), suggesting that the repression mediated by these three Ago proteins is insignificant. This finding is consistent with previous reports that in mammals, Ago2 represses target genes efficiently because of its slicer activity.^4,5^ By contrast, Ago2 had a similar activity as the other Ago proteins in repressing miRNA targets in which the guiding miRNAs were not fully complementary to the targets (**Figure 1a**). Thus, designing siRNA/shRNA to be Ago2-specific might achieve maximum functionality while avoiding saturation of Ago1, 3, and 4, which can execute endogenous miRNA function without Ago2, and would therefore minimize disturbance of the endogenous miRNA repression function.

It has been reported that transfection of siRNA can globally perturb gene expression because of impaired effectiveness of endogenous miRNA repression.^26^ It has previously been shown that the large numbers of conventional shRNA can saturate key components of miRNA machinery, such as Exportin5 to cause substantial toxicity.^30,31,32^ Our study suggested that ectopically expressed shRNA can also compromise the endogenous miRNA function to cause loss of the repression of target gene similarly to siRNA transfection, possibly because of the competition for Ago proteins (**Figure 3**). Comparing to the relatively limited number of unintended off-target genes caused by the siRNA or shRNA, which has been intensively studied, the compromised function of endogenous miRNA by siRNA or shRNA that can globally perturb gene expression, should be a more serious concern in using siRNA and shRNA for research or therapy, which has been largely ignored currently. Our study showed that designing shRNA to be Ago2-specific can effectively resolve this concern.

The newly learned lesson from miR-451 biogenesis made it possible to design Ago2-specific siRNA/shRNA by simply mimicking miR-451. Surprisingly, native miR-451 were active in all four Ago-expressing cells probably owing to their propensity to form dimers because of the mismatch at position 1 and G:U wobble pairs at positions 6 and 18 (**Figure 6a**), which can be processed by Dicer and loaded into all Ago proteins and therefore be less Ago2-specific. For both synthetic siRNA oligos and U6 promoter-driven shRNAs, stabilizing the stem by removing the mismatches can improve the Ago2 specificity (**Figures 2** and **6**), suggesting that it might be a better structure than the native miR-451 structure for designing Ago2-specific siRNA and shRNA.

By comparing the functionality of 15 pairs of shRNAs with either a miR-451-like structure with perfect match stem or a conventional shRNA structure, we showed that miR-451-like shRNAs have different sequence preferences than conventional shRNAs. Interestingly, we also observed that miR-451-like shRNAs tended to have higher functionality in most cases in which there was significantly different functionality (**Figure 5**). The higher functionality of miR-451-like shRNAs in general might be explained by reduced competition from Ago1, 3, and 4, as it has been reported that these other Ago proteins compete with Ago2 for siRNA loading.^33^ In the case of conventional shRNA, shRNA-derived duplex will be loaded into Ago1, 3, and 4 at a similar rate as into Ago2,^20^ therefore reducing the effective concentration of the duplex available to Ago2, while miR-451-like shRNAs can only be processed by Ago2, minimizing the competition from other Ago proteins.

How exactly siRNA duplexes are loaded into Ago proteins is not clear. According to the “rubber band” model, Hsc70/Hsp90 can change the conformation of Ago proteins like a stretched rubber band that opens the siRNA/miRNA duplex.^34^ While this pathway appears to apply to all Ago proteins, another loading mechanism in which Ago2 can cleave the fully complementary passenger strand removed by the C3PO complex is specific for Ago2.^7,8^ Thus, if a siRNA duplex is stabilized sufficiently, the stretched Ago proteins might not be able to open the duplex, but the duplex might be able to be loaded into Ago2 by the cleavage pathway. As previously shown by Petri *et al*.^29^, four LNAs are sufficient to stabilize the siRNA duplex for specific loading into Ago2. However, our data showed that a four-locked nucleic acid siRNA duplex can still efficiently be utilized by Ago1, 3, and 4 (**Figure 7**). The reason might be that Ago1, 3, and 4 can load siRNAs even though the efficiency of loading might be much lower than Ago2, because, in our system, only individual Ago proteins were expressed and did not compete with other Ago proteins.

## Materials and methods

*Cells, RNA oligos, transfection, and luciferase assays.* RNA oligos were ordered from Sigma (St Louis, MO) or IDT (Coralville, IA), and locked nucleic acid-stabilized oligos were ordered from AnaSpec. The sequences are listed in **Supplementary Table S1**.

ES cells-expressing Ago1–4 were cultured as described previously. shRNA constructs (0.1 µg) or siRNA (2 pmol) and 0.1 µg of the psiCHECK vectors containing the target sequences were reverse transfected with 4 × 10^4^ cells per well in 96-well plates. Dual-Glo luciferase assays were performed the next day per the manufacturer's instructions.

293FT cells (Invitrogen, Carlsbad, CA) were cultured according to the manufacturer's instructions. The day before transfection, the cells were trypsinized and diluted to 10^5^ cells per ml and seeded in 96-well plates in a volume of 100 µl per well. The shRNA construct (0.1 µg) and 0.1 µg of the psiCHECK2 plasmid harboring the shRNA target regions to be tested were cotransfected with lipofectamine 2000 (Invitrogen) per the manufacturer's instructions but with modifications. Dual-Glo luciferase assays (Promega, Madison, WI) were performed 1 day after transfection.

*Constructs.* All the DNA oligos are listed in **Supplementary Table S2**. DNA oligos were obtained from IDT and Sigma. For shRNAs constructs, the oligos were annealed and inserted into pLL3.7 at *HpaI* and *XhoI* sites. For the luciferase reporter assay, the oligos were annealed and inserted into psiCHECK2 at *XhoI* and *NotI* sites. The psiCHECK2 harboring full-length c-myb 3′ *UTR* was a kind gift from Dr. Changchun Xiao's lab.

*Real-time PCR for small RNA expression.* RT-PCR-based real-time PCR has been described previously.^35^ Briefly, 300 ng of the small-RNA fraction was polyadenylated with a poly(A) tailing kit (Epicentre Biotechnologies, Charlotte, NC) according to the manufacturer's instructions. The reaction was extracted with phenol/chloroform, and precipitated with ethanol. The poly(A)-tailed small RNAs were annealed with an anchored oligo dT (miR RT oligo dT) and transcribed with the Superscript III first-chain synthesis kit (Invitrogen) according to the manufacturer's instructions. cDNAs were amplified with SYBR green 2× mix (Takara, Bio USA, Madison, WI) for 15 seconds at 95 °C and 30 seconds at 60 °C for 45 cycles, followed by a thermal denaturing step to generate melting curves to verify amplification specificity. The primers used in this assay are listed in **Supplementary Table S3**.

*Quick cooling step for transforming the miR-451-like RNA oligo dimers to monomers.* The RNA oligos were diluted to 5 µmol/l and heated to 95 °C for 3 minutes then quickly cooled in an ice-water bath with shaking for 5 minutes.

*Statistical analysis.* Student's *t*-test (two-tailed, assuming equal variances in all experimental data sets) was used to compare two groups of independent samples.

**Supplementary material** **Table S1.** RNA oligos used in the study. “+” indicates the LNA position. **Table S2.** DNA sequences inserted into psiCHECK2 and pLL3.7 vectors. **Table S3.** Primer sequences for measuring small RNA expression.

## Supplementary Material

Supplementary Table S1 RNA oligos used in the study. “+” indicates the LNA position.

Supplementary Table S2 DNA sequences inserted into psiCHECK2 and pLL3.7 vectors.

Supplementary Table S3 Primer sequences for measuring small RNA expression.
