# Supplementary material for: Designing Ago2-specific siRNA/shRNA to Avoid Competition with Endogenous miRNAs
Source: Mol Ther Nucleic Acids. 2014 Jul 15;3(7):e176–. doi: 10.1038/mtna.2014.27 (PMC4121517; doi:10.1038/mtna.2014.27)
Supplement: Supplementary Table S3 — Primer sequences for measuring small RNA expression. [file mtna201427x3.pdf]

Supplemental Table 3

miR RT oligo dT	GCGAGCACAGAATTAATACGACTCACTATAGGT(20)VN
Universal reverse primer	GCGAGCACAGAATTAATACGAC
miR-150 forward primer	CTCCCAACCCTTGTACCAG
miR-451 forward primer	AACCGTTACCATTACTGAG
